# Pharmacokinetics of PEGylated Gold Nanoparticles: In Vitro—In Vivo Correlation

**DOI:** 10.3390/nano12030511

**Published:** 2022-02-01

**Authors:** Tibor Dubaj, Katarina Kozics, Monika Sramkova, Alena Manova, Neus G. Bastús, Oscar H. Moriones, Yvonne Kohl, Maria Dusinska, Elise Runden-Pran, Victor Puntes, Andrew Nelson, Alena Gabelova, Peter Simon

**Affiliations:** 1 Institute of Physical Chemistry and Chemical Physics, Faculty of Chemical and Food Technology, Slovak University of Technology in Bratislava, Radlinskeho 9, 812 37 Bratislava, Slovakia; alena.manova@stuba.sk (A.M.); peter.simon@stuba.sk (P.S.); 2 Cancer Research Institute, Biomedical Research Center SAS, v.v.i., Dubravska cesta 9, 845 05 Bratislava, Slovakia; Katarina.Kozics@savba.sk (K.K.); monika.sramkova@savba.sk (M.S.); alena.gabelova@savba.sk (A.G.); 3 Campus UAB, Catalan Institute of Nanoscience and Nanotechnology (ICN2), CSIC and BIST, Bellaterra, 08193 Barcelona, Spain; neus.bastus@icn2.cat (N.G.B.); oscarhernando.moriones@icn2.cat (O.H.M.); victor.puntes@icn2.cat (V.P.); 4 Fraunhofer Institute for Biomedical Engineering IBMT, 66280 Sulzbach, Germany; yvonne.kohl@ibmt.fraunhofer.de; 5 Health Effects Laboratory, NILU-Norwegian Institute for Air Research, 2007 Kjeller, Norway; mdu@nilu.no (M.D.); erp@nilu.no (E.R.-P.); 6 Institució Catalana de Recerca i Estudis Avançats (ICREA), 08010 Barcelona, Spain; 7 Vall d’Hebron Institut de Recerca (VHIR), 08032 Barcelona, Spain; 8 School of Chemistry, University of Leeds, Leeds LS2 9JT, UK; A.L.Nelson@leeds.ac.uk

**Keywords:** gold nanoparticles, human cell lines, pharmacokinetics, PBPK model, IVIVE

## Abstract

Data suitable for assembling a physiologically-based pharmacokinetic (PBPK) model for nanoparticles (NPs) remain relatively scarce. Therefore, there is a trend in extrapolating the results of in vitro and in silico studies to in vivo nanoparticle hazard and risk assessment. To evaluate the reliability of such approach, a pharmacokinetic study was performed using the same polyethylene glycol-coated gold nanoparticles (PEG-AuNPs) in vitro and in vivo. As in vitro models, human cell lines TH1, A549, Hep G2, and 16HBE were employed. The in vivo PEG-AuNP biodistribution was assessed in rats. The internalization and exclusion of PEG-AuNPs in vitro were modeled as first-order rate processes with the partition coefficient describing the equilibrium distribution. The pharmacokinetic parameters were obtained by fitting the model to the in vitro data and subsequently used for PBPK simulation in vivo. Notable differences were observed in the internalized amount of Au in individual cell lines compared to the corresponding tissues in vivo, with the highest found for renal TH1 cells and kidneys. The main reason for these discrepancies is the absence of natural barriers in the in vitro conditions. Therefore, caution should be exercised when extrapolating in vitro data to predict the in vivo NP burden and response to exposure.

## 1. Introduction

The unique physicochemical properties of gold nanoparticles (NPs) make them an emerging platform for a wide range of pharmaceutical and biomedical applications, especially in diagnosis and therapy. Therefore, a comprehensive understanding of their pharmacokinetics is essential for predicting their potential efficacy (i.e., to achieve sufficient exposure in target tissues while minimizing side effects) and safety in biomedical applications. Demand for methods allowing fast and reliable assessment of NPs’ biodistribution, fate, and possible toxicity is high. The “gold standards” for pharmacokinetic and toxicological studies are experiments on mice or rats and interspecies extrapolation. Disadvantages of these in vivo studies are rather apparent: ethical issues, high costs, and the time-consuming nature. These aspects of in vivo studies are further aggravated considering the large variety of NPs differing in their physicochemical characteristics (e.g., size, charge, chemical composition, and surface modification). Thus, in vivo data suitable for assembly and parameterization of the physiologically-based pharmacokinetic (PBPK) model remain relatively scarce. Cell-based in vitro experiments are less expensive, faster, and easier to perform compared to in vivo studies and also conform to the “3Rs” (Replace, Reduce, and Refine) principle. For these reasons, the European Commission strongly requests and promotes the development of alternative test methods and their application in the field of hazard and risk assessment of nanomaterials or chemicals in general.

Most preliminary toxicological and preclinical studies are now carried out in vitro using cell lines. However, one of the limitations of these in vitro experiments is the lack of biological barriers present in living organisms. Cells of different origins are directly exposed to NPs dispersed in a cultivation medium; however, in living organisms, some cells may never come in direct contact with NPs [1]. Cell response to exposure is then monitored by various cellular and molecular methods. Even by intravenous administration, primarily used in biomedical applications, the NPs first have to escape the immune cells before crossing the endothelial cells of the capillaries to reach the tissues. Moreover, in the case of NP biodistribution, additional factors, such as corona formation, greatly determine their biological fate [2,3]. However, despite the lack of whole organism complexity, the model cell lines may provide valuable insight into the mechanisms of NP uptake and potential interactions with cell counterparts under strictly defined conditions, allowing to minimize inter-individual variability. To simulate the in vivo conditions, more sophisticated in vitro models have been developed that mimick, for example, blood–brain [4,5], lung [6,7], gut [8], skin [9,10], and placenta [11,12] barriers using cell culture inserts, microfluidic systems, or other cell–medium configurations. Although they are promising tools, due to their small physical dimensions, only a limited number of cells can be obtained, which hinders reliable quantification of NPs in cells required for kinetic description of the internalization process. Thus, in vivo studies will not be replaced by in vitro studies in the foreseeable future because even the most sophisticated body-on-a-chip platforms are far from the complexity of real organisms. Over recent years, there has been a clear trend of translating the results from in vitro and in silico studies into in vivo for hazard and risk assessment of NPs (also called “in vitro to in vivo extrapolation”, IVIVE) [13,14,15,16].

For low-molecular substances, in vitro data are already routinely used to parameterize whole-body PBPK models [17,18] employing parameters, such as organ masses, blood flow rates, and enzyme activities, relevant to the organism’s real physiology. Tissues and organs are treated as compartments linked together by the central compartment (blood), usually assuming classical first-order reaction kinetics.

Among engineered nanomaterials, gold NPs are relatively well explored in terms of their pharmacokinetics and ultimate fate [19]. It appears that biodistribution of gold NPs is largely determined by their size rather than their surface modification: >10-nm gold NPs tend to accumulate in the liver and spleen irrespective of ligands present [20,21], while smaller particles (under 6 nm) undergo fast renal clearance with essentially no accumulation in organs [22]. However, to the best of our knowledge, studies using the same type of gold NPs and investigating their kinetics of uptake/clearance in vitro and in vivo are missing. Such studies are especially suitable to examine the limitations of correlating in vitro and in vivo data.

In this study, we evaluated the reliability of extrapolating the results from in vitro experiments to predict in vivo biodistribution of gold NPs. To minimize the effect of confounding factors, the same 13-nm gold NPs coated with polyethylene glycol (PEG-AuNPs) were used in both experiments. Several human cell lines generally used as surrogate models of particular in vivo tissues were employed and rats were injected with PEG-AuNPs via single tail-vein administration. Rats are considered more suitable models than mice in extrapolating the pharmacokinetics of gold NPs to humans [23]. A non-mechanistic model for NP translocation was applied to in vitro cell cultures and the resulting parameters were then utilized for predicting the biodistribution in vivo. Finally, the simulated biodistribution curves were compared with data published in our previous study [24].

## 2. Materials and Methods

### 2.1. Gold Nanoparticles 

Polyethylene glycol (PEG)-coated gold NPs (PEG-AuNPs) with a core size of 10.5 ± 0.8 nm and hydrodynamic diameter of 13.1 ± 3.0 nm were synthesized and characterized in depth using various physicochemical methods. Basic physicochemical characteristics of PEG-AuNPs have already been published [24] and can be found in the Supplementary Materials (SEM image, Figure S1; UV/Vis spectra, Figure S2; size and zeta potential distribution curves, Figure S3). The same stock of PEG-AuNPs was used in both in vitro and in vivo experiments. The microbial analysis did not determine any detectable levels of endotoxin contamination (<0.25 EU/mL) within the PEG-AuNPs solution (N01563935). 

### 2.2. Cell Lines

The human lung epithelial carcinoma cells A549 (ATCC^®^ CCL-185™), the human lung bronchial epithelial cells 16HBE (SCC150, Merck, Darmstadt, Germany), and the human hepatocarcinoma cells Hep G2 (ATCC^®^ HB-8065™) were cultured in Roswell Park Memorial Institute (RPMI) medium supplemented with L-glutamine (4 mM), penicillin (100 U/mL), streptomycin (100 μg/mL), and 10% (*v*/*v*) fetal calf serum (FCS). The human renal proximal tubule epithelial (TH1) cells purchased from Kerafast Inc. (Boston, MA, USA) were cultivated in Dulbecco’s Modified Eagle Medium (DMEM) medium with high glucose (4.5 g/L) supplemented with penicillin (100 U/mL), streptomycin (100 μg/mL), and 10% FCS. The human bronchial epithelial cells 16HBE (SCC150, Merck, Darmstadt, Germany) were cultured in DMEM/Nutrient Mixture F-12 Ham medium supplemented with penicillin (100 U/mL), streptomycin (100 μg/mL), and 10% (*v*/*v*) FCS. All cell lines were cultivated at 37 °C in a humidified atmosphere of 5% CO_2_.

### 2.3. Cell Treatment

The A549, 16HBE, Hep G2, and TH1 cells were seeded at several P. dishes. After reaching confluence of 75–80%, cells were exposed to PEG-AuNPs at a concentration of 5 µg mL^−1^. This working concentration was prepared as previously described [24,25]. The kinetics of PEG-AuNPs uptake into individual cell lines, representing surrogate in vitro models of particular organs, was investigated at several time-point intervals (1 h, 2 h, 4 h, 6 h, 9 h, 16 h, 24 h, and 48 h) after single-dose application. At a particular sampling time, the medium was sucked off, and cells were washed twice with phosphate buffer solution (PBS). Then, cells were detached from the bottom of the P. dishes by trypsinization, pooled (3 P. dishes per time interval), spun down, and the pellet was frozen and kept at −20 °C until analysis by graphite furnace atomic absorption spectrometry (GFAAS). The conditions of samples’ mineralization and subsequent GFAAS analysis were the same as those used for in vivo study [24]. 

In addition, the 16HBE cells were exposed to PEG-AuNPs (5 µg mL^−1^) under dynamic flow conditions using a microfluidic in vitro platform described in [26]. The flow rate of the medium was 100 µL h^−1^.

### 2.4. Biodistribution and Accumulation of PEG-AuNPs In Vivo 

The experiment was carried out on six to eight-week-old male Wistar rats. The design of the in vivo experiment and PEG-AuNPs administration have already been published [24]. In brief, male Wistar rats (b. w. 210–230 g) were injected via tail vein with a single dose of PEG-AuNPs (0.70 mg kg^−1^) suspension and sacrificed after 1 h, 4 h, 24 h, 7 days, and 28 days post-exposure. Subsequently, blood, liver, spleen, kidneys, and lungs were collected and stored at −20 °C until their analysis by GFAAS. 

### 2.5. Quantification of PEG-AuNPs in the Cells, Blood, and Organs 

Graphite furnace atomic absorption spectrometry (GFAAS) was employed to quantify the internalized amount of gold in individual cell lines, blood, and organs as previously described [24]. In brief, microwave sample digestion system Multiwave GO (Anton Paar, Graz, Austria) equipped with high-pressure PTFE vessels was used to mineralize cell cultures and rat tissue after HNO_3_–HCl digestion. The temperature program of digestion was as follows: slow heating (20 min) from room temperature to 170 °C, 10 min holding at 170 °C, and 10 min cooling.

A high-resolution atomic absorption spectrometer AA700 (Analytik Jena AG, Jena, Germany), equipped with graphite furnace atomizers, was used to quantify elemental gold in biological samples. Measurements were carried out at 242.8 nm using integrated absorbance summed over three pixels. All measurements were performed using pyrolytically coated graphite tubes with an integrated PIN platform (Analytik Jena, Part No. 407-A81.026). All reagents were of an analytical grade of the highest purity available. Throughout the experiments, water from the NANOpure system (Wilhelm Werner GmbH, Germany) was utilized. As a stock solution for AAS calibration, a standard 1.000 gL^−1^ gold solution was employed. The limit of detection (LOD) was determined to be lower than 0.125 μg L^−1^. The amount of gold was expressed as µg of gold per g of cells or tissue.

### 2.6. Data Treatment and Statistics

In vivo data are given as mean values ± SEM; in vitro data correspond to a result of pooled experiments performed in triplicate at each time interval. The model for NPs translocation was fitted to in vitro data using the non-linear least squares method using OriginPro 9.1; the resulting parameters are presented as best estimate ± SE. The PBPK model was simulated using the SimBiology toolbox (Matlab R2018b).

## 3. Results and Discussion

### 3.1. In Vitro Pharmacokinetic Study

The amounts of internalized gold in individual cell lines found at different sampling times are listed in Table 1. Notable differences can be seen in the quantity of gold determined in individual cell lines: the highest amount of gold, which further increased over exposure time, was identified in renal TH1 cells, followed by A549 lung cells. 

Surprisingly, the lowest gold amount was detected in the Hep G2 cells. Moreover, in contrast to other cell lines, the quantity of internalized gold exhibited larger scatter over the whole sampling period.

### 3.2. Kinetic Analysis of Experiments with Cell Cultures

Both in vitro and in vivo data were fitted with a reasonable minimum number of adjustable parameters. The translocation of PEG-AuNPs (internalization and expulsion) can be viewed as first-order processes with rate constants *k*_in_ and *k*_out_, respectively:

(1) $$\mathrm{NPs}\;\mathrm{in}\;\mathrm{medium}\;\overset{k_{\mathrm{in}}}{\underset{k_{\mathrm{out}}}{⇄}}\;\mathrm{NPs}\;\mathrm{in}\;\mathrm{cells}.$$

The resulting rate equation with respect to the total amount of PEG-AuNPs internalized in cells is

(2) $$\frac{dA_{C}}{dt}=m_{C}\frac{dc_{C}}{dt}=k_{\mathrm{in}}c_{M}m_{C}-k_{\mathrm{out}}c_{C}m_{C},$$

where *A*_C_ is the NPs amount internalized in cells, *m*_C_ is the total mass of the cells, and *c*_C_ and *c*_M_ is the concentration of NPs in cells and medium, respectively. During the experiment, the cells are growing under limited resources; their mass can be approximated by first-order growth kinetics as

(3) $$m_{C}=m_{C,\;\mathrm{eq}}[1-\exp(-k_{g}(t+t_{0}))],$$

where *m*_C,eq_ is the final mass of the cell culture, *k*_g_ is the growth rate constant, and *t*_0_ is growth time prior to NPs exposure. The concentration of NPs in the surrounding medium, *c*_M_ can be obtained from the mass balance as (*A*_total_ − *A*_C_)/*m*_M_. Since the NPs are in great excess compared to the internalized amount (*A*_total_ >> *A*_C_), *c*_M_ can be considered constant. Solving the Equation (2) with *m*_C_ expressed using Equation (3) and assuming *A*_C_(0) = 0 yields

(4) $$A_{C}(t)=\frac{A_{\mathrm{total}}k}{k_{\mathrm{out}}(k_{g}-k_{\mathrm{out}})}(k_{g}-k_{\mathrm{out}}+k_{\mathrm{out}}e^{-k_{g}(t+t_{0})}-k_{g}e^{-k_{\mathrm{out}}t}+k_{\mathrm{out}}e^{-k_{\mathrm{out}}t}-k_{\mathrm{out}}e^{-k_{\mathrm{out}}t-k_{g}t_{0}}),$$

where *k* = *k*_in_(*m*_C,eq_/*m*_M_). The fitting model defined by Equation (4) only contains two adjustable parameters (*k*_in_ and *k*_out_) since *k*_g_, *A*_total_, *t*_0_ and *m*_C,eq_ can be determined independently from *A*_C_. For the in vitro model, it is considered that the cells grow during the exposure so that their mass increases with time. In case of a flow-through setup used for 16HBE cells where the cells are continuously perfused with fresh medium, Equation (4) retains its form, however, with *A*_total_*k* replaced by *c*_M_*k*_in_*m*_M_ where *m*_M_ is the total mass (or volume) of the medium used during the run. 

**Figure 1:**
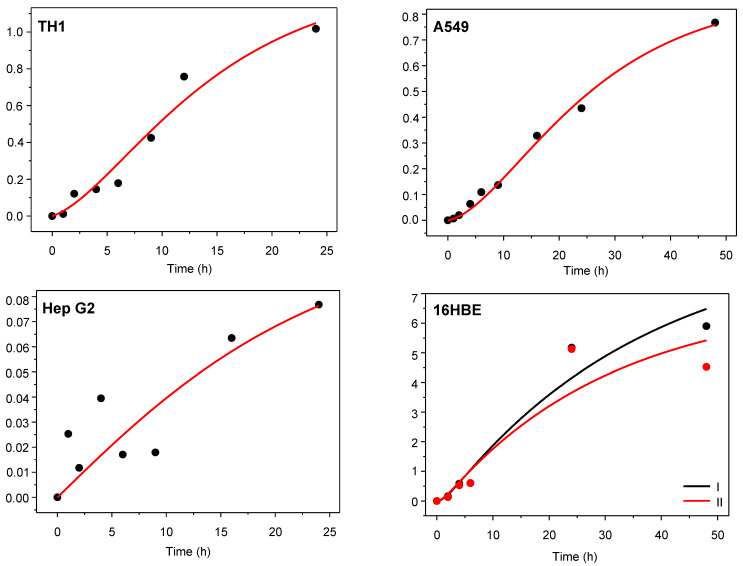
Results of fitting Equation (4) to the determined amount of Au internalized in various cell lines. The vertical axis is the total amount of internalized PEG-AuNPs in μg.

The model for PEG-AuNPs uptake represented by Equation (4) was fitted by the non-linear least-squares method to data from Table 1; the resulting curves are depicted in Figure 1. As it can be seen from Table 2, a good or at least satisfactory fit quality was obtained: the coefficient of determination (*R*^2^) ranges from 0.67 for Hep G2 to 0.99 for A549 cells. The best-fitting values of translocation rate constants in both directions (*k*_in_ and *k*_out_) were used to calculate the cells:medium partition coefficient, *R*_c:m_, for each cell line under study.

The main disadvantage of such a non-mechanistic model resides in the unclear physical meaning of its parameters. In this case, the values of *R*_c:m_ only reflect the overall affinity of a particular cell line towards PEG-AuNPs accumulation, irrespective of the underlying transport mechanisms. However, they can be used to predict concentration-time curves with *R*_c:m_ as a surrogate to the partition coefficient in vivo. 

### 3.3. In Vivo Pharmacokinetic Study

**Figure 2:**
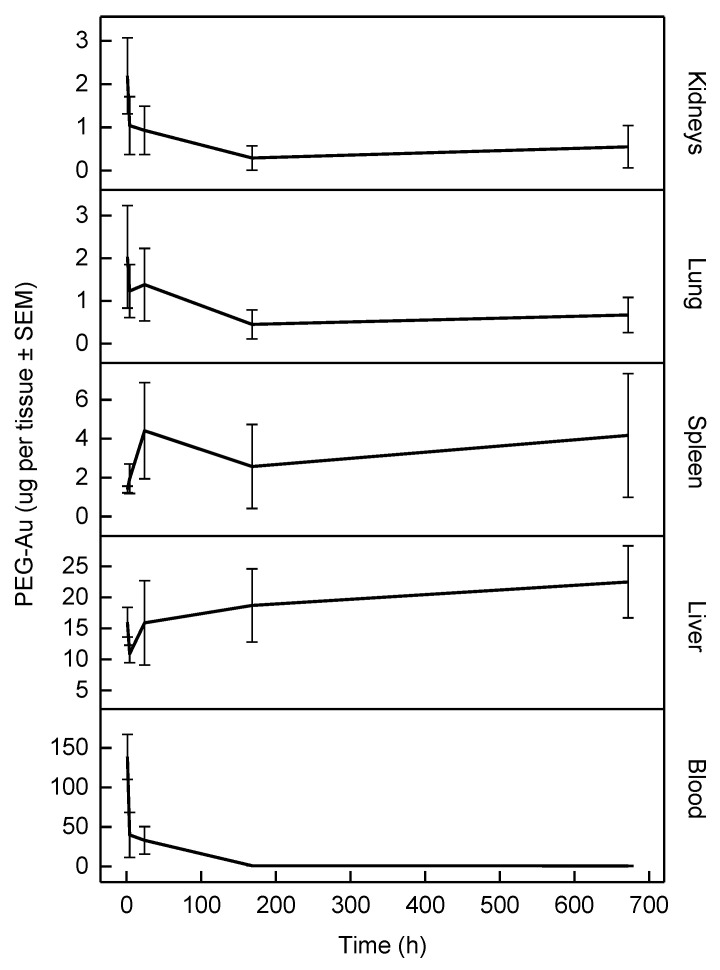
Time course of PEG-Au NPs burden in individual tissues; data obtained from Kozics et al. [24]. The error bars correspond to ± SEM.

The kinetics of PEG-AuNP distribution and accumulation in rats’ blood, liver, spleen, kidney, and lungs after a single tail-vein injection has been previously published [24]. The time course of PEG-AuNPs burden in individual tissues is depicted in Figure 2. It is evident that compared to AuNPs of similar diameter coated with triphenylphosphine mono-sulfonate [21,27] or dextran [28], the surface modification with PEG prolonged the circulation time with a blood half-life of 57 h (non-PBPK two-compartment model). Nonetheless, in line with the above-referenced studies, the liver and spleen were the primary sites of NPs accumulation. The dose recovery after 1 h in the blood and four tissues analyzed in our study was 89%; however, this decreased significantly to 30% and 16% after 4 h and 672 h, respectively. Thus, the NPs were either excreted via bile or transported into other tissues (the remainder).

### 3.4. Transfer of Parameters from In Vitro to In Vivo

The most straightforward way for translating the *k*_in_ and *k*_out_ values obtained from in vitro experiments into the PBPK-related parameters is to use the perfusion rate-limited PBPK model. The applicability of this type of PBPK on NP biodistribution is often questioned; however, perfusion rate-limited PBPK models require the smallest number of adjustable parameters. In this model, the mass-balance equation for tissues is

(5) $$\frac{dA_{t}}{dt}=Q_{t}\left(c_{\mathrm{bl}}-\frac{c_{t}}{R_{t\text{:}\mathrm{bl}}}\right)-CL_{t}c_{t},$$

where *A*_t_ is the amount of NPs accumulated in the tissue, *c*_bl_ is the NPs’ concentration in arterial blood, *Q*_t_ is the regional blood flow, *CL*_t_ is the clearance (for excreting organs), and *R*_t:bl_ is the tissue:blood partition coefficient describing the equilibrium between the concentration of NPs. The model assumes that the leaving (venous) blood and the tissue are in equilibrium with respect to their NPs’ concentration:

(6) $$\mathrm{NPs}\;\mathrm{in}\;\mathrm{blood}\;\overset{{k^{\prime }}_{\mathrm{in}}}{\underset{{k^{\prime }}_{\mathrm{out}}}{⇄}}\;\mathrm{NPs}\;\mathrm{in}\;\mathrm{tissue},$$

where ${k^{\prime }}_{\mathrm{in}}$ and ${k^{\prime }}_{\mathrm{out}}$ are the corresponding first-order translocation rate constants. In other words, the model assumes that the equilibrium indicated in the scheme (6) is fast. The perfusion rate-limited model has a great advantage of relying only on physiologically determined parameters (*V_t_* and *Q_t_*) and a thermodynamic (equilibrium-related) parameter *R*_t:bl_. Thus, the tissue:blood partition coefficient in Equation (5) can be viewed as

(7) $$R_{t\text{:}\mathrm{bl}}=\frac{{k^{\prime }}_{\mathrm{in}}}{{k^{\prime }}_{\mathrm{out}}}.$$

The in vitro surrogate models of in vivo tissues lack the barriers provided by capillary walls, which affects the values of translocation rate constants. However, assuming that the barrier has proportionally the same effect on both ${k^{\prime }}_{\mathrm{in}}$ and ${k^{\prime }}_{\mathrm{out}}$, their ratio is then similar to that of *k*_in_ and *k*_out_ obtained from in vitro experiments. Thus, obtaining *k*_in_ and *k*_out_ from in vitro experiments allows to parameterize the PBPK model using Equation (5) and correlate the resulting concentration-time curve with those obtained from in vivo study.

**Figure 3:**
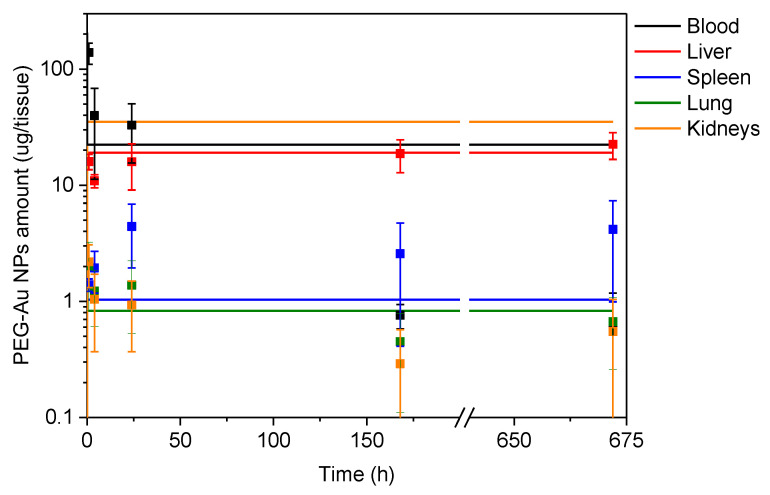
Gold amounts determined in vitro in different cell lines (lines) and in vivo in blood and individual organs (points). The physiological parameters used are shown in Table 3. The cell–tissue assignments were as follows: liver, spleen = Hep G2, lung = 16HBE, and kidneys = TH1.

The simulated curves together with in vivo data are depicted in Figure 3. Due to the absence of in vitro data from splenic cells, for the simulation purposes, the spleen was treated with the same *R*_c:m_ value as the liver (Hep G2 cells). From Figure 3, it can be seen that compared to in vivo data, the perfusion rate-limited model equilibrates more quickly with PEG-AuNPs amount varying only slightly over the sampling time under study. The two main sites of PEG-AuNPs accumulation observed in vivo (liver and spleen) are pretty close to the values predicted from the in vitro experiments. The highest discrepancy was found for renal TH1 cells, where the predicted values are almost two orders of magnitude higher than those observed in vivo. The human TH1 cells are a valuable surrogate model of the proximal renal convoluted tubules, the most vulnerable kidney compartment due to the concentration of toxicants [29]. However, before the primary filtrate reaches the proximal tubules in vivo, it has to pass through the glomerular basal membrane (GBM), a natural barrier for NPs with a cut-off diameter of approximately 6 nm [30]. The active endocytic machinery of proximal renal tubules efficiently internalizes filtered proteins, including NPs [31]. The absence of a natural barrier on the one hand and the increased endocytic activity, on the other hand, can explain the observed discrepancy between in vitro and in vivo data. Similarly, A549 cells also exhibit high affinity towards NP accumulation, while 16HBE cells show the opposite. The lack of barrier properties in A549 cells has already been demonstrated for 50-nm silica NPs [32]. Thus, in vivo results for lungs were compared with 16HBE rather than A549. Results for 16HBE cells are close to those for in vivo, thus reflecting their good barrier properties already reported in literature [33].

When analyzing the distribution of NPs either in vivo or in vitro, it is tempting to assemble a pharmacokinetic model tailored to a specific mechanism for NP uptake and elimination, including protein corona formation and immune cells. Protein corona can critically affect their recognition by the innate immune system and their biological effects. Some models have already been developed to describe the NP-protein interactions during corona formation [35]. To minimize this effect, the starting point of NPs’ dilution was to mix NPs in stock solution with an equal volume of 100-% FBS. Using this approach, the same protein corona should always be formed. However, equally important is to consider the role of mononuclear phagocytes: they affect biodistribution and clearance of NPs, and mediate inflammatory and immunological biological responses [36]. There were several attempts to include the mononuclear phagocytic system (MPS) as a part of the PBPK model for NP biodistribution, e.g., [37,38]. However, despite the resulting parameters having more clear physical meaning, such an approach introduces additional body-specific or NPs-specific parameters (number or concentration of phagocytes, their uptake rate constant, and uptake capacity). None of these values are known a priori and are usually treated as adjustable parameters, thus contributing to model overparameterization and ill-conditioning. On the other hand, the application of perfusion rate-limited models to PEG-AuNP biodistribution is frequently disputed since NPs can hardly be regarded as “small molecules” whose uptake and biodistribution are limited by regional blood flow through the tissue. Therefore, just as in vitro *R*_c:m_, the in vivo *R*_t:bl_ values represent a rather unpredictable amalgamate effect of several processes. In the case of NPs, the membrane-limited PBPK models are generally more suitable [39], which consider the active (and practically one-way) internalization of NPs by phagocytic cells (macrophages, monocytes, and dendritic cells) as the main route of NP uptake into tissues, and the total NP burden is then correlated with the number of phagocytes in the corresponding tissue. Clearly, the cell lines used for in vitro experiments cannot simulate the active uptake of NPs by phagocytes. Even though there are in vitro studies on NP toxicity with macrophage cell lines [40,41,42], currently there is no sensible way for translating their results into a PBPK model. The perfusion rate-limited model used in this study completely neglects these aspects of NP biodistribution and can be viewed as a special case of the membrane-limited model with the membrane permeability coefficient set to one and NPs’ absorption rate of phagocytes set to zero for all organs.

## 4. Conclusions

Mathematical modeling helps to improve the understanding of NPs’ behavior in biological systems with respect to physicochemical and physiological parameters. Therefore, integrating mathematical modeling with experimental measurement of the kinetics, efficacy, and toxicity of NPs will become increasingly important for their biomedical translatability. Our results of in vitro–in vivo correlation of PEG-AuNP distribution and pharmacokinetics suggest that cell lines can, in some cases, provide a sensible model for NPs’ internalization in living organisms. The in vivo PEG-AuNP biodistribution assessed in rats was compared with predicted distribution using a non-mechanistic model applied to in vitro cells. The internalization and exclusion of PEG-AuNPs were modeled as first-order rate processes with the partition coefficient describing the overall tendency of NP accumulation. However, care should be taken when translating results for organs/tissues with natural barriers whose absence in vitro may lead to greatly overestimated NP burden when compared to in vivo distribution. Another caveat of in vitro models based on pure cell lines resides in the absence of macrophage-mediated uptake, which may greatly affect the fate of NPs in living organisms.

## Figures and Tables

**Table 1:** Internalized amounts of 13-nm PEG-AuNPs determined by GFAAS (expressed as μg g^−1^).

Cell Line	Sampling Time (h)							
	1	2	4	6	9	16	24	48
TH1	4.87	7.49	11.75	13.71	19.79	26.35	32.51	160.96 ^1^
A549	1.2	4.07	5.81	9.95	10.74	14.18	16.68	30.94
Hep G2	1.48	0.56	2.06	0.8	0.85	2.14	3.2	–
16HBE I ^2^	–	1.07	4.02	3.80	–	–	3.07	2.83
16HBE II ^2^	–	0.79	3.50	3.77	–	–	3.04	2.33

^1^ Value excluded due to possible cell disruption/disintegration. ^2^ Flow-through experiments at flow rate 100 μL h^−1^ (two replicates).

**Table 2:** First-order rate constants for translocation of 13-nm PEG-Au NPs (best estimate ± SE).

Cell Line	*R* ^2^	*k*_in_ (h^−1^)	*k*_out_ (h^−1^)	*R*_c:m_ = *k*_in_/*k*_out_
TH1	0.96	1.28 ± 0.30	0.171 ± 0.056	7.48
A549	0.99	0.400 ± 0.033	0.0598 ± 0.0078	6.69
Hep G2	0.67	0.051 ± 0.024	0.063 ± 0.060	0.810
16HBE I	0.95	0.40 ± 0.60	0.66 ± 1.03	0.606
16HBE II	0.86	0.50 ± 1.45	0.94 ± 2.79	0.532

**Table 3:** Organ masses and regional blood flow rates for rat obtained from [34].

Organ/Tissue	Mass ^1^	Blood Flow ^2^
Blood	6.40	–
Liver	3.66	18.3
Spleen	0.20	0.85
Lung ^3^	0.50	2.10
Kidneys	0.73	14.1

^1^ Expressed as the percentage of body weight. ^2^ Expressed as the percentage of cardiac output (0.110 L min^−1^). ^3^ Bronchial circulation only.

## Data Availability

No other data except a Supplemental file published at MDPI website are available.

## Supplementary Materials

The following supporting information can be downloaded at: https://www.mdpi.com/article/10.3390/nano12030511/s1, Figure S1: TEM image of PEG-AuNPs; Figure S2: UV/Vis spectra of PEG-AuNPs colloidal solution; Figure S3: DLS spectra and zeta potential distribution curves of of PEG-AuNPs colloidal solution.
