# Supplementary material for: Pharmacokinetics of PEGylated Gold Nanoparticles: In Vitro—In Vivo Correlation
Source: Nanomaterials (Basel). 2022 Feb 1;12(3):511. doi: 10.3390/nano12030511 (PMC8838925; doi:10.3390/nano12030511)
Supplement: Supplementary file 1 [file nanomaterials-12-00511-s001.zip › nanomaterials-1557485-supplementary.pdf]

Supplementary Material

Pharmacokinetics of PEGylated Gold Nanoparticles: In Vitro—In Vivo Correlation

Tibor Dubaj [1,*], Katarina Kozics [2], Monika Sramkova [2], Alena Manova [1], Neus G. Bastús [3], Oscar H. Moriones [3], Yvonne Kohl [4], Maria Dusinska [5], Elise Runden-Pran [5], Victor Puntes [3,6,7], Andrew Nelson [8], Alena Gabelova [2] and Peter Simon [1]

1 Institute of Physical Chemistry and Chemical Physics, Faculty of Chemical and Food Technology, Slovak University of Technology in Bratislava, Radlinskeho 9, 812 37 Bratislava, Slovakia; alena.manova@stuba.sk (A.M.); peter.simon@stuba.sk (P.S.)
2 Cancer Research Institute, Biomedical Research Center SAS, v.v.i., Dubravska cesta 9, 845 05 Bratislava, Slovakia; Katarina.Kozics@savba.sk (K.K.); monika.sramkova@savba.sk (M.S.); alena.gabelova@savba.sk (A.G.)
3 Catalan Institute of Nanoscience and Nanotechnology (ICN2), CSIC and BIST, Campus UAB, Bellaterra, 08193 Barcelona, Spain; neus.bastus@icn2.cat (N.G.B.); oscarhernando.moriones@icn2.cat (O.H.M.); victor.puntes@icn2.cat (V.P.)
4 Fraunhofer Institute for Biomedical Engineering IBMT, 66280 Sulzbach, Germany; yvonne.kohl@ibmt.fraunhofer.de
5 Health Effects Laboratory, NILU-Norwegian Institute for Air Research, 2007 Kjeller, Norway; mdu@nilu.no (M.D.); erp@nilu.no (E.R.-P.)
6 Institució Catalana de Recerca i Estudis Avançats (ICREA), 08010 Barcelona, Spain
7 Vall d'Hebron Institut de Recerca (VHIR), 08032 Barcelona, Spain
8 School of Chemistry, University of Leeds, Leeds LS2 9JT, United Kingdom; A.L.Nelson@leeds.ac.uk;
* Correspondence: tibor.dubaj@stuba.sk

1 MATERIALS AND METHODS

1.1 Chemicals

Gold(III) chloride trihydrate $HAuCl_4{\cdot}3H_2O$ (99%), Sodium citrate tribasic hydrate $HOC(COONa)(CH_2COONa)_2$ (99%) and PEG-thiol (mercaptopolyethylene glycol monomethyl ether, $CH_3O(CH_2CH_2O)_nCH_2CH_2SH$, M_n = 5000) and other chemicals were purchased from Sigma–Aldrich (Merck Life Science, Madrid, Spain). Milli-Q water was used in all experiments. All glassware was cleaned with acetone, rinsed with deionized water, and stored at 150 °C before use and all reagents were used as received without further purification.

1.2 Physicochemical Characterization

UV/Vis Spectroscopy: UV-Visible spectra were acquired with an Agilent Cary 60 UV-Vis Spectrophotometer (Agilent Technologies, Santa Clara, CA, USA). 1 mL of the NPs was placed in a quartz cuvette, and spectral analysis was performed in the 300 nm to 800 nm range at room temperature.

Electron Microscopy: particles were visualized using a JEOL JEM 1010 transmission electron microscope (JEOL, Kyoto, Japan) operated at 20 kV in transmission mode STEM.

The samples were prepared by drop-casting 10 μL of the sample onto a piece of ultrathin carbon-coated 200-mesh copper grid (Ted Pella, Inc.) and left to dry in air. TEM images of the prepared colloidal NPs were used for the size distribution measurements using Image J software. The size of at least 300 particles was measured for each sample, and the average size and the standard distribution were obtained.

Dynamic Light Scattering (DLS): The hydrodynamic size and surface charge of the AuNPs before and after conjugation with a thiol-PEG molecule were determined by Dynamic Light Scattering (number and intensity mean) and Laser Doppler Anemometry, respectively, using a Zetasizer Nano ZS instrument (Malvern Instruments, Malvern, UK) equipped with a light source wavelength of 532 nm and a fixed scattering angle of 173°. Aliquots (1.0 mL) of the colloidal NP solutions were placed into specific plastic cuvettes and the software was arranged with the parameters of refractive index and adsorption coefficient of gold, and solvent viscosity of water at 25 °C. Each value was the average of at least 3 independent measurements. All measurements used the Smoluchowski model.

1.3 Synthesis of the PEGylated gold nanoparticles (AuNPs)

LPS-free sterile gold particles (Au NPs) were synthesized following the well-reported kinetically controlled seeded growth method via the reduction of $HAuCl_4$ by sodium citrate[1]. Briefly, a solution of 2.2 mM sodium citrate in Milli-Q water (150 mL) was heated with a heating mantle in a 250 mL three-necked round-bottomed flask for 15 min under vigorous stirring. A condenser was utilized to prevent the evaporation of the solvent. After boiling had commenced, 2.5 mL of $HAuCl_4$ (25 mM) was injected. The color of the solution changed from yellow to bluish-grey and then to soft pink in 10 min. Immediately after the synthesis of the Au seeds and in the same reaction vessel, the reaction was cooled until the temperature of the solution reached 90 °C. Then, 1 mL of a $HAuCl_4$ solution (25 mM) was injected. After 30 min, the reaction was finished. This process was repeated twice. The resulting particles are coated with negatively charged citrate ions and hence are well suspended in H_2O. Later, the NPs were directly conjugated with a solution of Mercaptopolyethylene glycol monomethyl ether, having a final concentration of 2 μM of thiol-PEG stirring at 600 r.p.m for about 24 h. Finally, the NPs were washed with pure water by centrifugation 10.000g for 20 min and were concentrated five times to achieve a concentration of 0.396 mg Au /mL and ~4,05 $\times 10^{14}$ NPs/mL respectively.

2 RESULTS

2.1 Characterization of the PEGylated gold nanoparticles (PEG-AuNPs)

<table>
<tr><td>Solvent:</td><td colspan="3">LPS-free sterile milli Q-water</td></tr>
<tr><td>Appearance: (concentrated colloidal water solution of PEG-AuNPs)</td><td colspan="3">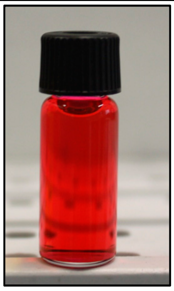</td></tr>
<tr><td rowspan="3">Concentration:</td><td>$\approx 4.05 \times 10^{14}$</td><td colspan="2">NPs/mL</td></tr>
<tr><td>2.01×10^{-3}</td><td colspan="2">Au mmol/mL</td></tr>
<tr><td>0.396</td><td colspan="2">Au mg/mL</td></tr>
<tr><td>Safety requirements for handling:</td><td colspan="3">wear gloves and protective googles</td></tr>
<tr><td>Surface functionalisation:</td><td colspan="3">PEG-Thiol, MW ≈ 5000 (Ω-end = SH, α-end = OCH)</td></tr>
<tr><td>Sample purification:</td><td colspan="3">Washed and dispersed in LPS-free water. The process is repeated twice.</td></tr>
<tr><td>Dispersion protocol:</td><td colspan="3">When stored for a long period of time Au NPs may sediment at the bottom of the vial, which is especially true for larger particle sizes. Prior to use, re-suspend the sedimented particles by swirling until a homogenous solution is obtained.</td></tr>
<tr><td colspan="3">Transmission Electron Microscopy (TEM), d</td><td>10.5 ± 0.83 nm</td></tr>
<tr><td colspan="3">Dynamic Light Scattering (DLS), d</td><td>Peak 1 (intensity): 38.8 ± 12.8 nm
Peak 1 (number): 13.0 ± 3.02 nm
PdI: 0.2014 ± 0.019</td></tr>
<tr><td colspan="3">UV/Vis Spectroscopy</td><td>Absorption peak at 519.2 nm</td></tr>
<tr><td colspan="3">Zeta Potential (in water at pH 6.80)</td><td>−9.12 ± 1.58 mV</td></tr>
</table>

2.2 Transmission Electron Microscopy (TEM)

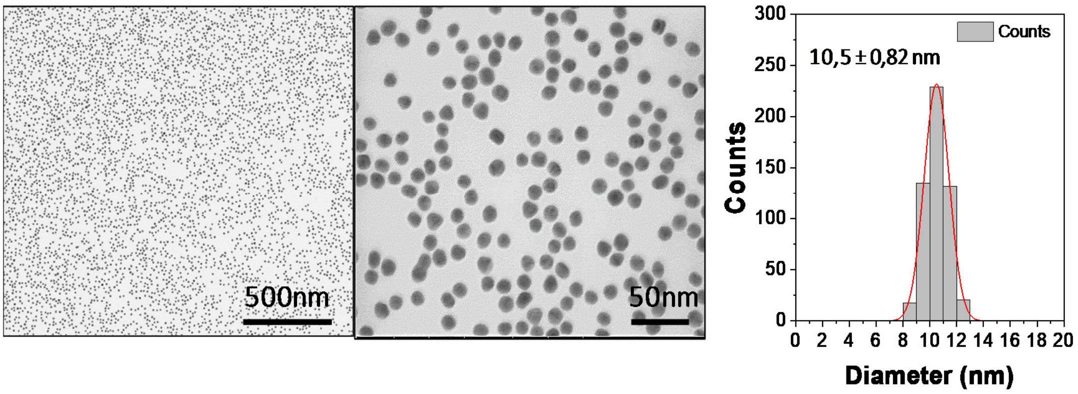

Figure S1. Transmission electron microscopy images of the PEGylated gold NPs and the calculated size distribution after measuring the diameter of *n* = 533 particles by Image J software. The mean particle size is 10.5 ± 0.82 nm.

2.3 UV/Vis Spectroscopy

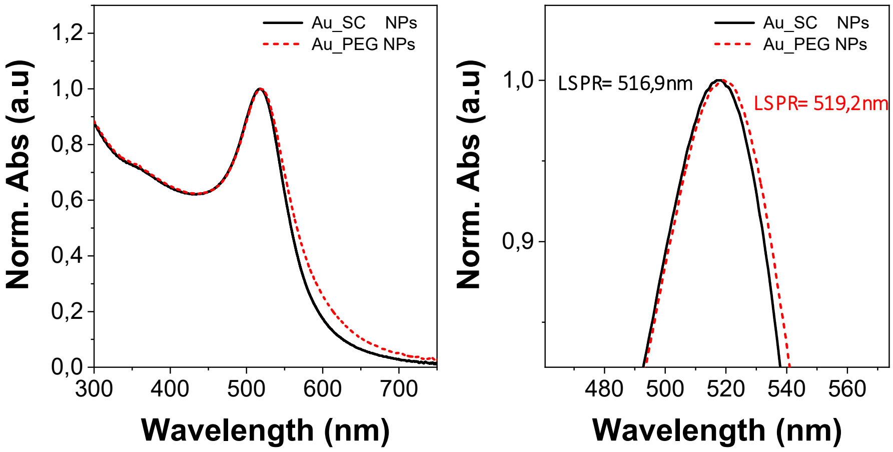

Figure S2. Normalized UV/Vis spectra of the colloidal solution of Au_SC and Au_PEG NPs. The spectra were taken at 1:10 dilution in pure water at neutral pH. As shown in the spectra The localized plasmon LSPR peak of the Au can be observed at wavelength of 516.9 nm and is red-shifted to a 519.2 nm after the conjugation with PEG.

2.4 Dynamic Light Scattering (DLS), *d* (nm)

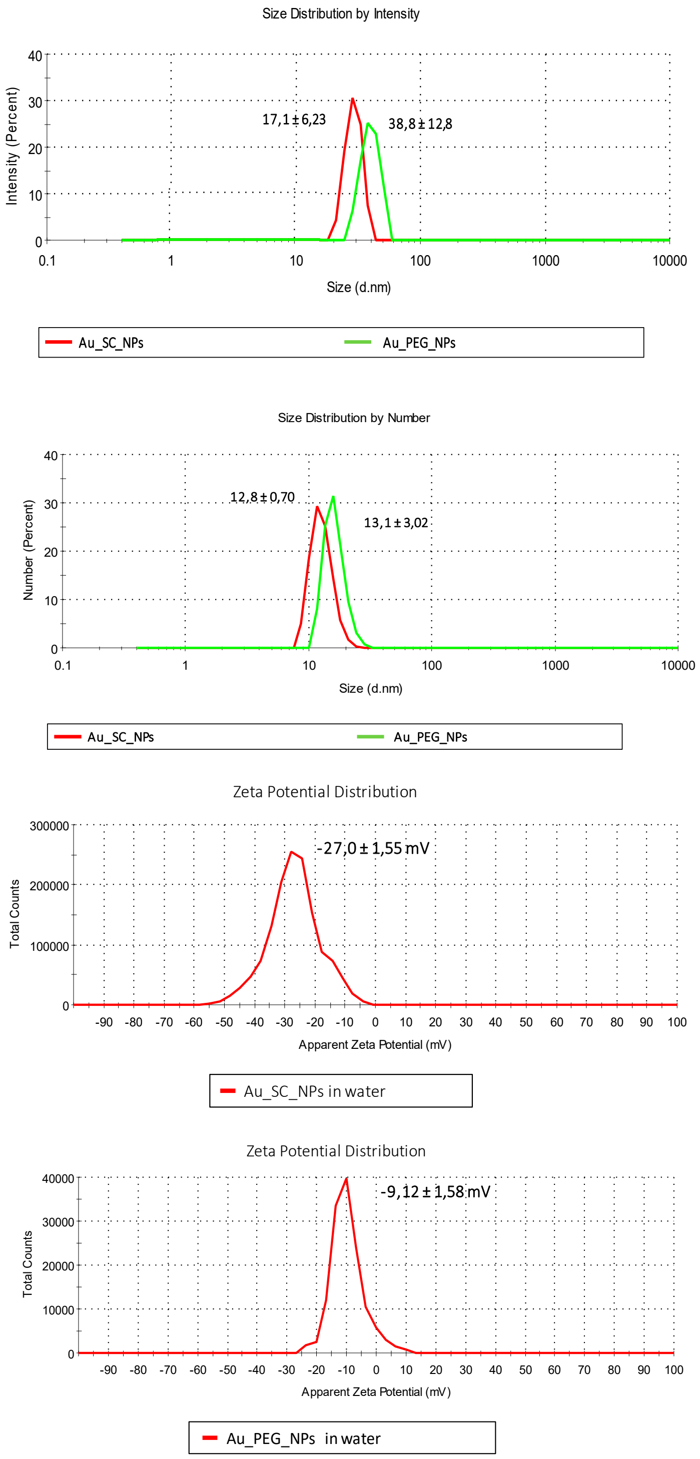

Figure S3. DLS spectra and zeta potential distribution curves of the colloidal solution of Au_SC and Au_PEG NPs.

Reference

1. Bastús, N.G., Comenge, J., Puntes, V. Kinetically Controlled Seeded Growth Synthesis of Citrate-Stabilized Gold Nanoparticles of up to 200 nm: Size Focusing versus Ostwald Ripening. *Langmuir* **2011**, *27(17)*, 11098–11105, https://doi.org/10.1021/la201938u.
